# Mesenchymal stromal cell-based therapy for cartilage regeneration in knee osteoarthritis

**DOI:** 10.1186/s13287-021-02689-9

**Published:** 2022-01-10

**Authors:** Xiao-Na Xiang, Si-Yi Zhu, Hong-Chen He, Xi Yu, Yang Xu, Cheng-Qi He

**Affiliations:** 1 Department of Rehabilitation Medicine, West China Hospital, Sichuan University, Chengdu, 610041 Sichuan People’s Republic of China; 2 School of Rehabilitation Sciences, West China School of Medicine, Sichuan University, Chengdu, 610041 Sichuan People’s Republic of China; 3 Key Laboratory of Rehabilitation Medicine in Sichuan Province, West China Hospital, Sichuan University, Chengdu, 610041 Sichuan People’s Republic of China; 4 Rehabilitation Medicine Centre, West China Hospital, Sichuan University, Chengdu, 610041 Sichuan People’s Republic of China

**Keywords:** Mesenchymal stromal cell, Osteoarthritis, Exosome, Regeneration, Cartilage

## Abstract

Osteoarthritis, as a degenerative disease, is a common problem and results in high socioeconomic costs and rates of disability. The most commonly affected joint is the knee and characterized by progressive destruction of articular cartilage, loss of extracellular matrix, and progressive inflammation. Mesenchymal stromal cell (MSC)-based therapy has been explored as a new regenerative treatment for knee osteoarthritis in recent years. However, the detailed functions of MSC-based therapy and related mechanism, especially of cartilage regeneration, have not been explained. Hence, this review summarized how to choose, authenticate, and culture different origins of MSCs and derived exosomes. Moreover, clinical application and the latest mechanistical findings of MSC-based therapy in cartilage regeneration were also demonstrated.

## Background

Osteoarthritis is a common and disabling condition that represents substantial health and socioeconomic costs with notable implications for the individuals affected and healthcare systems [1, 2]. Global percentage change of years lived with disability in counts between 2006 and 2016 was 31.5% [3]. Knee osteoarthritis (KOA) is the most prevalent subtype of osteoarthritis [4] that shows symptoms with pain, swell, stiffness, and loss of mobility mainly in the aging and obese populations [5]. The pathogenesis of KOA is complicated, not only associated with the “wear and tear,” which is called mechanical stress [6]. Microenvironmental and genetic factors interact during deterioration that ultimately leads to degeneration of articular cartilage, intraarticular inflammation with synovitis, and changes in subchondral bone [7, 8]. Extracellular matrix (ECM) produced and secreted by chondrocytes and synovial fluid secreted by fibroblast-like synoviocytes are the two main important substances to maintain the microenvironment [9, 10]. Nonetheless, chondrocytes constitute only 2% of cartilage volume [11, 12] and may the first be activated by inflammatory signals originating from synovium or subchondral bone [13], which alter the balance between synthesis and degradation of ECM and cause the limited potential for self-regeneration of articular cartilage. Recommended treatment options range from non-care currently limited to pain control and merely improve the regeneration of articular cartilage in KOA.

Cell-based therapy and novel approaches using mesenchymal stromal cells (MSCs) or exosomes secreted by MSCs are presented as alternative cell-based sources to chondrocytes, which show potential for cartilage regeneration in KOA [14, 15]. The International Society for Cell & Gene Therapy refers MSCs as a bulk population with notable secretory, immunomodulatory, and homing properties. The minimal criteria include being plastic adherent, expressing specific surface markers, and capable of in vitro differentiation into adipocyte, chondrocyte, and osteoblast lineages [16]. MSCs and secreted exosomes (Exos) maintain therapeutical potentials for cartilage regeneration, including balancing metabolic activity of cartilage and the chondrogenic differentiation, which has been reported in animal studies and gaining positive outcomes in the clinic [17–19]. However, a common standard for cell selection and preparation for studies and applications of MSCs is not yet available. Moreover, the mechanism of MSCs and MSC-Exos for KOA is not demonstrated clearly yet. This paper briefly describes situations associated with currently used methods for cell selection and preparation, followed by a review of the existing literature on MSC-based cell therapy for cartilage regeneration in KOA.

## MSCs and MSCs-exosomes: cell selection and preparation

### Cell selection

Stromal cells having multipotent differentiation potentials with regenerative capacity can be generally classified into two groups: embryonic stromal cells and adult stromal cells [20]. Embryonic stromal cells contain cells from the term placenta, amniotic fluid, and umbilical cord. Adult stromal cells have been identified within most of the tissues or organs, and the following sources have been applied for treating KOA, such as bone marrow (BM) [21, 22], trabecular bone [23], adipose tissue (AT) [19], synovial fluid [24], synovium [25], and peripheral blood [26]. Stromal cells from different sources have different differentiation capacities, different clinical benefits, and cultural characteristics [27]. Hence, cell source is an important consideration for successful outcomes in mesenchymal stromal cell therapies and the common sources include bone marrow, adipose, synovial fluid, and synovium. According to the number of studies, bone marrow-derived MSCs (BM-MSCs) could be the predominant cell source, followed by adipose tissue-derived MSCs (AT-MSCs).

Exosome is a specific extracellular vesicle ranged from 30 to 150 nm diameter [28] with a density of between 1.1 and 1.2 g/mL [29], found in multiple types of cell [30] and extracellular fluids, such as plasma [31], synovial fluid [32], urine [33], amniotic fluid [34], saliva [35], cerebrospinal fluid [36], breast milk [37], and tears [38]. MSC-Exos transfer bioactive lipids, nucleic acids (DNA, mRNAs, and non-coding RNAs) [39], and proteins between cells to elicit biological responses (gene-regulation [40], proliferation, apoptosis [41], immunomodulation [42], and so on) in recipient cells [43]. Different MSC-Exos have heterogeneity, even extracellular RNA extracted from exosomes and non-vesicles derived by the same cell have heterogeneity [44].

### Phenotypic analysis

Phenotypic analysis confirms the expression of various MSCs-related surface markers. Most MSCs are positive for cluster of differentiation (CD)73 (5'­nucleotidase) [45–51], CD90 (Thy­1 membrane glycoprotein) [48–54], CD105 (endoglin) [32, 50, 51, 55–57], CD44 (hyaluronan receptor) [48, 49, 58, 59], and lack expressions for CD34 (hematopoietic progenitor cell antigen) [32, 55, 56, 60, 61], CD14 (myeloid cell-specific leucine-rich glycoprotein) [60–63], CD45 (protein tyrosine-phosphatase) [64–68], and HLA-DR (human leukocyte antigens class II DR) [62, 68, 69]. Individual markers include CD146 (S-endo1, melanoma cell adhesion molecule, Muc18, or glycerin) [65–67, 70], CD29 (integrin β­1) [45–47, 52, 53, 64–66], CD49e (integrin α­5), CD54 (intercellular adhesion molecule 1), CD106 (vascular cell adhesion molecule) [63], CD146 (melanoma cell adhesion molecule) [32, 55, 56, 67, 70], CD166 (activated leukocyte cell adhesion molecule) [63, 67], CD271 (low-affinity nerve growth factor receptor) [32, 46, 47, 55, 56, 65–67], SSEA-4 (stage-specific embryonic antigen-4) [45], Notch 1 (neurogenic locus notch homologue protein 1), HLA-ABC (human leukocyte antigens, histocompatibility complex class I molecules) [71, 72], and Stro­1 (stromal antigen 1) [68].

Besides CD44, CD73, CD90, CD105, some protein markers have the potentials to be new and specific markers [73]. Stro-1 and CD271 are cell membrane single-pass type I proteins that translocate from the endoplasmic reticulum to the cell membrane in response to the depletion of intracellular calcium. However, it is unclear whether Stro-1 expression correlates with multipotency [46]. SSEA-4 is an embryonic stem cell marker, and CD146 is detected on perivascular cells around venules [69]. Erdogan et al. reported that AT-MSCs in New Zealand rabbits did not express CD73 and CD90 [58], while Chen et al. detected the expression of CD90 [59]. Some markers appeared already at the optic vesicle stage but did not remain highly expressed in the later differentiation stage [74].

As for Exos, they are characterized by the expression of endosomal markers, including tetraspanins (CD9, CD63, CD81, and CD82) due to endosomal origin [32, 64, 68, 75–77], whereas TSG101 (tumor suppressor gene 101), an endosomal sorting complex required for transport-related protein specific for micro-vesicle body formation, is not specifically expressed in exosomes [32]. The common surface marker profile of MSCs and MSC-exosomes is shown in Table 1.

**Table 1:** Surface markers on mesenchymal stromal cells and exosomes

Species	Source	Positive antigens	Negative antigens	References
Human	Bone marrow	CD13, CD29, CD44, CD71, CD90, CD106, CD120a, CD124, CD271, CD146, Stro-1, SSEA-4	CD14, CD34, CD45	[65, 66]
Rabbit	Bone marrow	CD29, CD73, CD105, CD146	CD34, CD45	[45]
Rat	Bone marrow	CD29, CD44, CD90	CD34, CD11, CD45	[52, 53]
Mice	Bone marrow	Sca-1, CD29	CD45, CD11b	[64, 65]
Human	Adipose	CD13, CD29, CD44, CD73, CD90, CD105, CD271, CD146	CD31, CD34, CD45, Stro-1, SSEA-4	[46, 47]
Rabbit	Adipose	CD29, CD44, α-SMA, CD90	CD34, CD45	[58, 59]
Rat	Adipose	CD44, CD73, CD90	CD34, CD45, CD11b	[48, 49]
Mice	Adipose	CD29, CD105	CD34, CD45	[57]
Human	Synovial fluid/synovium	CD13, CD73, CD90, CD105, Stro-1, SSEA-4, CD146	CD11b, CD14, CD19, CD34, CD45, CD79b, CD271, HLA-DR	[32, 55, 56]
Human	Blood	CD29, CD73, CD90, CD105, CD146, CD166	CD45, Stro-1, SSEA-4, CD271	[67]
Horse	Blood	CD73, CD90, CD105, CD146		[70]
Human	Term placenta	CD29, CD44, CD73, CD90, CD105, SSEA-4	CD11b, CD14, CD19 CD31, CD34, CD45, Stro-1, HLA‐DR, CD271	[60, 61]
Human	Amniotic fluid	CD73, CD90, CD105	CD31, CD34, CD45	[54]
Human	Umbilical cord	CD73, CD90, CD105	CD11b, CD14, CD19, CD34, CD45, HLA-DR, CD271, SSEA-4	[62]
Human	Trabecular bone	CD90, CD73, CD105, CD166, CD106, CD146	CD14, CD19, CD34, CD45	[63]
Human	BM-MSC-Exos	CD9, CD81, TSG101	Calnexin	[68]
Rabbit	BM-MSC-Exos	CD9, HSP70		[75]
Rat	BM-MSC-Exos	CD63, CD81, TSG101	Calnexin	[76]
Mice	BM-MSC-Exos	CD63, CD81, syntenin 1, TSG101		[64, 77]
Human	AT-MSC-Exos	CD9, CD63, CD73, CD81, CD90, CD146, TSG 101, HLA-ABC	Calnexin, CD45, HLA-DR	[68]
Human	SF-MSC-Exos	CD9, CD63, CD81, TSG101		[32]
Human	UC-MSC- Exos	CD63	Calnexin	[62]

*MSCs* Mesenchymal stromal cells, *BM* bone marrow, *AT* adipose tissue, *UC* umbilical cord, *SF* synovial fluid, *Exos* exosomes, *CD* cluster of differentiation, *SSEA-4* stage-specific embryonic antigen-4, *α-SMΑ* α-smooth muscle actin, *HLA* human leukocyte antigen, *TSG101* Recombinant Tumor Susceptibility Gene 101, *HSP70* heat-shock protein 70

### Culture

MSCs are spindle-shaped and adherent cells, capable of proliferation, self-renewal, and differentiating into cells of multi-lineage. One of the characteristic features of MSCs is adhering to tissue culture plastic and generating colonies when plated at low densities [78]. MSCs growing from individual foci, or colonies from the microscopic view, and these colonies generated from progenitor cells have been called the colony-forming unit fibroblast [79]. The ability of MSCs to undergo chondrogenic, osteogenic, and adipogenic differentiation has been reported in vitro and in vivo.

**Fig. 1:**
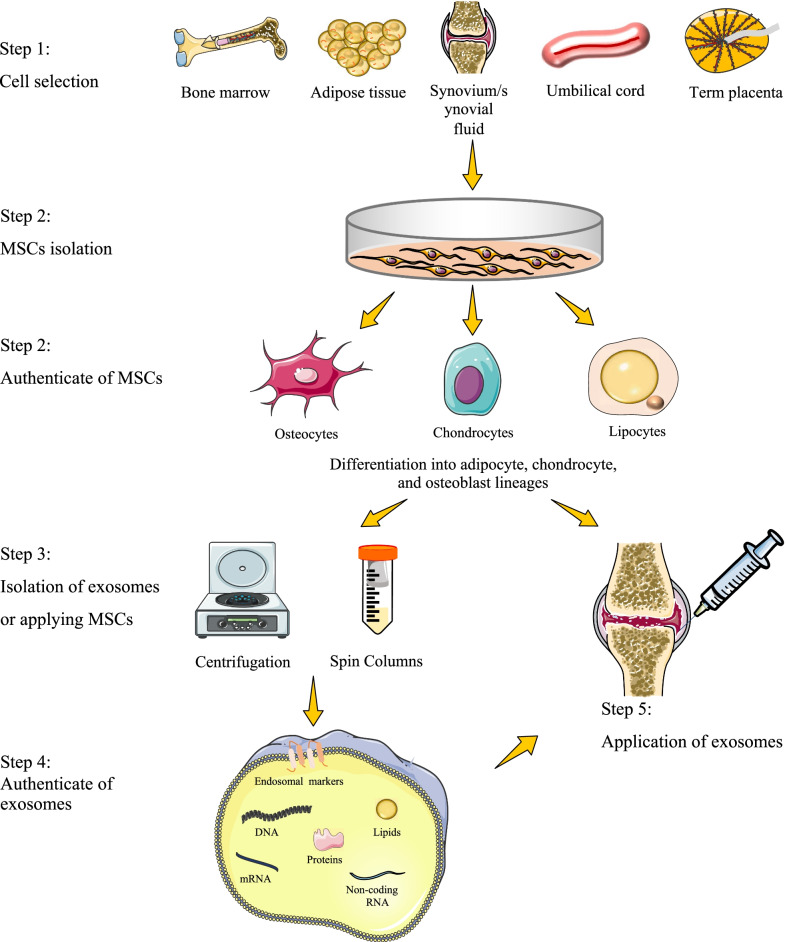
The flow diagram of applying MSC-based therapy. Firstly, choose the appropriate origin of MSCs, then isolate MSCs from other cells, and authenticate them. Inject the MSCs or isolate MSCs-derived exosomes by centrifugation or other methods, and then inject the exosomes after authentication. MSCs: mesenchymal stromal cells

Chondrogenic differentiation largely depends on the culture conditions. Mediators capable of promoting chondrogenesis, such as transforming growth factor-beta (TGF-β), have been elucidated using simplified in vitro models [80]. Recently, Yin et al. indicated that MSCs differentiated into mature chondrocytes after 21 days of co-culture with ECM-derived particles in a microgravity environment without exogenous TGF-β3 [81]. Chondrogenesis can be achieved either in 2-dimensional or 3-dimensional culture systems in vitro. The 3-dimensional culture system facilitates greater cell contacts and interactions of cells with the ECM, allowing cells to adapt to their native morphology [82]. Moreover, the efficiency of chondrogenesis tends to be lower in the 2-dimensional culture system. The scaffold-free 3-dimensional cultures provide a high-density cell culture environment and are commonly classified as pellet or micromass culture systems [83]. In general, the induced cartilage was more similar to hyaline cartilage in the micromass culture technique, while the pellet culture is more useful for clinical applications [84]. Moreover, platelet-rich plasma (PRP), MSCs, and chondrocytes co-culture would favor chondrogenesis without hypertrophic and pathologic responses [85]. Chondrocytes cultured with MSC-Exos enhanced proliferation and chondrogenesis [86]. The flow diagram of applying MSC-based therapy is presented in Fig. 1.

## Functions of MSC-based therapy for cartilage regeneration in KOA

### Impact on chondrocyte

Increased chondrogenesis, improved proliferation, reduced apoptosis, and maintenance of autophagy of chondrocytes are the main functions of MSC-based therapy on KOA. The chondrogenesis is primarily due to chondrogenic differentiation of MSCs triggered by some growth factors or enhanced activity of chondroprogenitors and chondrocytes stimulated by MSCs [87]. Moreover, human AT-MSCs showed a high chondrogenic potential supported by the increased expression of sex-determining region of Y chromosome-box transcription factor 9 (SOX9), as a chondrocyte precursor marker [88]. One study reported a reduced matrix synthesis and low proliferation rate of chondrocytes in the injured cartilage, while the restored proliferation ki67 enhanced after injecting human umbilical cord-derived MSCs [89]. Additionally, the proliferation rate of chondrocytes was increased while co-culturing with BM-MSCs [90]. In addition, synovial MSC-Exos have the potential to improve proliferation and migration of chondrocytes in vitro and in vivo [91]. TGF-β1-stimulated MSC-Exos increased the cell viability of the chondrocyte-restricted rat cell line [92]. Rat BM-MSC-Exos under hypoxic pretreatment promoted the proliferation and migration of chondrocytes [93].

Apoptosis of chondrocytes is another characteristic of the progression of OA. Chen et al. reported a notable decrease of the proportion of dUTP nick end labeling positive cells that indicated apoptosis in cartilage after injection with MSCs compared with the phosphate-buffered saline [94]. Apoptosis could be a potential therapeutic pathway for KOA [95]. Moreover, the apoptosis of chondrocytes was remarkably inhibited by coculturing with BM-MSCs under hypoxia [90]. Exos also have a positive effect on preventing cartilage damage. Interleukin (IL)-1β treatment inhibited cell viability and DNA synthesis activity, and enhanced apoptosis of chondrocytes. However, BM-MSC-Exos treatment promoted cell viability and DNA synthesis activity with a lower apoptosis rate [96]. Wu et al. also reported AT-MSC-Exos inhibited cell apoptosis and enhanced autophagy [97]. Autophagy can be detected combined with apoptosis in KOA. Autophagy serves as an adaptive response under environmental changes that maintain the survival of chondrocytes by preserving energy metabolism in cells. The levels of Beclin‐1 and microtubule‐associated protein light chain 3, as two vital proteins in the autophagic process, were detected to manifest a reduced autophagic activity in KOA animal model that treated with phosphate-buffered saline, whereas MSCs therapy maintained almost the same level as that of the normal group [94].

### Impact on the ECM

Regulating the balance of synthesis and catabolism of the ECM is essential to treat degenerative diseases, such as KOA. Matrix metalloproteinases (MMPs) refer to a family of zinc-dependent ECM remodeling endopeptidases that degrade the ECM. On the other hand, the tissue inhibitors of MMPs (TIMPs) are important regulators of ECM turnover, tissue remodeling, and cellular behavior that inhibit the proteolytic activity of MMPs within the ECM. BM-MSCs balanced the ratio of MMP‐13 to TIMP‐1 in cartilage and reduced the expression of cartilage hypertrophic markers such as collagen (Col)-X, fibroblast growth factor receptors 1–3, parathyroid hormone-related protein, and MMP-13 [98]. Several studies reported the higher expression of gene Col 2α1 in KOA cartilage, which encoded the α-1 chain of Col II, after MSC-based therapy [24, 99]. Moreover, the expressions of a disintegrin and metalloproteinase with thrombospondin motifs-5 and MMP13 in cartilage were significantly downregulated after treating with human umbilical cord-derived MSCs [89]. Hyaluronan synthase-1 mRNA expression was upregulated in BM-MSCs after co-culture with chondrocytes from the KOA model, whereas hyaluronidase-1 was downregulated [100]. Chen et al. revealed that BM-MSC-Exos could promote the expression of Col II, SOX9, and aggrecan while negatively regulating the expression of chondrocyte hypertrophy markers MMP-13 in mouse models of post-traumatic KOA [101].

### Impact on the inflammatory cytokines

Inflammatory response plays an important role in the pathogenesis of KOA. The most important groups controlling the disease seem to be pro-inflammatory cytokines and anti-inflammatory cytokines, which have an antagonistic effect. The former mainly includes IL-1β, tumor necrosis factor‐α (TNF‐α), IL-6, IL-15, IL-17, and IL-18. Another is formed by TNF-stimulated gene 6, IL-4, IL-10, IL-13, IL-37, and others. IL-37 partly rescued IL-1β and impaired cartilage formation of MSCs. This effect contributed to a lower MMP3 expression and an increased ratio of Col-II/ Col-I without increasing hypertrophy markers [102]. Pro-inflammatory (M1) macrophages are associated with a high production of pro-inflammatory mediators such as TNF-α, IL-6, IL-1β, and IL-12, and are required for T cell activation. These cytokines induce destructive processes in chondrocytes manifesting a lower expression of Col-II and aggrecan synthesis [103].

Some researchers found decreased expressions of inflammatory and catabolic markers including IL-1β, TNF-α, and MMP13, after AT-MSCs injection [104], BM-MSCs injection [105], or induced pluripotent stromal cell-chondrocytes transplant in KOA model. However, no difference in Col-II and Col-I expression was found between transplanted cartilage and other groups [106]. One study determined that both IL-1β and TNF-α immunostaining in chondrocytes in the cartilage were significantly enhanced after human umbilical cord-derived MSCs treatment and reserved almost back to normal tissue. Additionally, umbilical cord-derived MSCs therapy also led to increased expression of anti-inflammatory factors, TNF-stimulated gene 6, and IL-1 receptor antagonist, in the articular chondrocytes [89]. Human AT-MSCs seem to adapt and respond better to both inflammatory stimuli and autologous protein solution than BM-MSCs in vitro [107]. Co-culture with AT-MSCs counteracted the IL-1β-induced mRNA upregulation of the MMP-3, MMP-13, TNF- α, and IL-6 in chondrocytes. Importantly, AT-MSCs increased the expression of the anti-inflammatory cytokine IL-10 in chondrocytes [108, 109].

As for treatment with Exos, injection of miR-9-5p-contained Exos alleviated the inflammation in KOA, which was evidenced by downregulated levels of inflammatory factors and reduced oxidative stress injury [110]. Lu et al. reported synovial MSC-Exos enhanced IL-1β-induced cell proliferation, whereas inhibited apoptosis and inflammation and the target relationship of miR-26a-5p and phosphatase and tensin homologue were predicted and confirmed [111]. Moreover, Zhe et al. investigated miR-26a-5p in human BM-MSCs exerted an alleviatory effect on the damage of the synovial fibroblasts [112].

### Impact on the immunity

Macrophages could play a crucial role in modulating inflammation during the pathogenesis of KOA via various secreted mediators. These cells can polarize to pro-inflammatory and anti-inflammatory (M2) phenotypes. One study exposed AT-MSCs to osteoarthritic synovial fluid for two days for determining the effect of their secretome on differentiation of monocytes into pro-inflammatory M1-like macrophages and mature dendritic cells, and the effect on T cell proliferation and expansion of T regulatory cells. The results suggest that the exposure of AT-MSCs upregulated the immunosuppressive factors that induce monocytes into the M2-like phenotype and inhibit differentiation of monocytes into mature dendritic cells. Only the secretome of exposed AT-MSCs was detected to inhibit proliferation of T cells and promote T regulatory cells expansion [113].

More than 240 micro-RNAs were found in AT-MSCs and accounted for most of the genetic message that protected chondrocytes and M2 macrophage polarizing. Ragni et al. [114] have confirmed an increased M2 phenotype marker CD163 and reduced the chondrocyte inflammation marker vascular cell adhesion molecule-1 on inflamed macrophages and chondrocytes. BM-MSCs-Exos and AT-MSCs-Exos have reported relieving KOA by promoting the phenotypic transformation of synovial macrophages from M1 to M2 [115, 116]. Moreover, TGF-β1-stimulated BM-MSC-Exos reduced pro-inflammatory factors by promoting M2 polarization of synovial macrophages [117].

### Impact on the mitochondrial function

Aging and exposure to stress would determine the chondrocyte phenotype in osteoarthritis and age-related mitochondrial dysfunction and associated oxidative stress might induce senescence in chondrocytes [118]. The mitochondrial transfer was found from BM-MSCs to osteoarthritis chondrocytes. One study showed an increased mitochondrial membrane potential when co-cultured with mitochondria from MSCs compared with chondrocytes without mitochondria transfer. The activity of mitochondrial respiratory chain enzymes and the content of adenosine-triphosphates were significantly improved [119].

### Impact on the paracrine effect

**Fig. 2:**
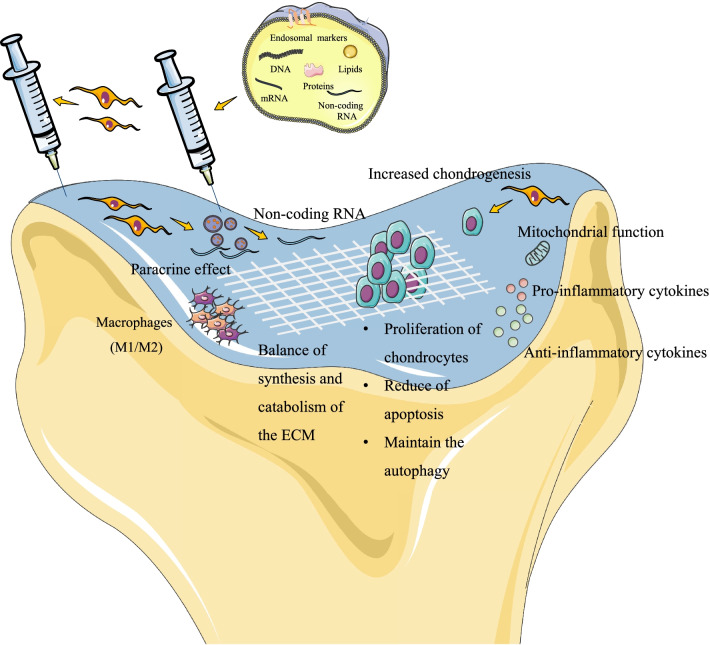
The functions of injections of MSCs or MSCs-derived exosomes. MSCs can protect cartilage by differentiation into chondrocyte lineages, affecting the chondrocytes, mediating mitochondrial function, regulating cytokines, balancing the synthesis and catabolism of the extracellular matrix (ECM), modifying immune reactions, and paracrine activity that might be involved with the secreted exosomes. Exosomes are small extracellular vesicles that include lipids, nucleic acids, and proteins. MSCs: mesenchymal stromal cells, ECM: extracellular matrix

Some researchers thought the paracrine effect of MSCs was mediated or performed by MSC-derived extracellular vesicles, while others support the induction of paracrine effect was independent of extracellular vesicles [120]. In general, the paracrine effect and Exos both represent cell-to-cell contact and biological information delivery. Extracellular vesicles have been traditionally classified into four subtypes, mainly based on their origins and sizes. MSC-Exos, as the smallest extracellular vesicles, have recently been suggested as a mechanism for their therapeutic potentials [121]. Figure 2 shows the functions of MSC-based therapy.

## Mechanisms in cartilage regeneration by MSC-based cell therapy

### Pathways or axis

Regulations of inflammation, immunoregulatory, and metabolic pathways and the expression of key molecules by the MSCs-based therapy have been revealed in recent research. The nuclear factor-kappaB (NF-κB) family plays crucial roles in various biological processes including mechanical processes, immunity, inflammation, and oxidative stress response, which could be activated by chemokines, pro-inflammatory cytokines, and degradation factors. NF-κB comprises five members: RelA (p65), c-Rel, RelB, NF-κB1 (p50), and NF-κB2 (p52) [122]. Yu et al. reported that the deletion of RelA in culturing BM-MSCs could increase the chondrogenic differentiation [123]. Treatment with human AT-MSCs significantly reduced the content of signal transducer and activator of transcription 3, which is a DNA-binding molecule that regulates the levels of many cytokines. Activation of signal transducer and activator of transcription 3 leads to increased pro-inflammatory cytokine production and immune responses [124]. BM-MSC-Exos inhibited mitochondrial-induced apoptosis in response to IL-1β, involving p38 mitogen-activated protein kinase (MAPK), extracellular regulated protein kinases (ERK), and protein kinase B pathways (AKT) [125].

Chronic inflammation may contribute to stress‐induced senescence of chondrocytes and cartilage degeneration during the progression. Treatment of chondrocytes with MSCs down‐regulated senescence markers induced by IL‐1β including senescence‐associated β‐galactosidase activity, accumulation of γH2AX foci, and morphological changes with enhanced formation of actin stress fibers. Additionally, the treatment reduced the activation of MAPK, ERK1/2, and p38 and to a lower extent the phosphorylation of c-jun N-terminal kinase 1/2 [126], which represents a classical inflammatory pathway. Besides this, inflammation is a prerequisite for the protective effect of AT-MSCs. Van Dalen et al. proved that local application of AT-MSCs in KOA joints led to rapid clustering of polymorphonuclear cells around AT-MSCs, while IL-1β stimulated this clustering which reduces the pro-inflammatory activity of the polymorphonuclear cells in vitro [127].

In human chondrocytes, oxidative stress may lead to DNA damage and senescence. In addition, reactive oxygen species (ROS) production is important in signaling pathways activated by IL-1β in chondrocytes. As oxidative stress is a key process in the induction and maintenance of senescence, Platas et al. investigated the effects of AT-MSCs on protein modification by ROS. In chondrocytes from the KOA model, IL-1β quickly induced the production of ROS and enhanced levels of 4-hydroxy-2-nominal-modified proteins, whereas AT-MSCs reduced the level of them [126]. In addition, human AT-MEC-Exos reduced the production of oxidative stress in OA chondrocytes stimulated with IL-1β and resulted in an upregulation of peroxiredoxin 6 [128].

Cell proliferation activated by AT-MSCs in KOA rabbits may be specifically regulating the glycogen synthase kinase-3β (GSK-3β)/cyclin D1/cyclin-dependent kinase (CDK)4/CDK6 pathway. It reduced the elevated serum level of cartilage oligomeric matrix protein, blocked increases in the mRNA, and protein expression of GSK3β while decreasing the mRNA and protein expression of cyclin D1/CDK4 and cyclin D1/CDK6 in cartilage [129]. Wu et al. demonstrated BM-MSCs increased the levels of the ECM proteins Col-II and SOX9 and decreased chondrocyte apoptosis and inflammation by upregulating the mediators of the autophagy phosphatidylinositol 3 kinase/AKT/mammalian target of rapamycin (mTOR) pathway [130].

### Key transcription factors

Key factors included TGFBI/BIGH3 (TGF-β-induced gene product-h3), bone morphogenetic proteins (BMPs), Nanog, and Oct4, which have important functions in cell adhesion, migration, proliferation, and apoptosis. TGFBI is a chondroprotective factor, released by MSCs and an anabolic regulator of cartilage homeostasis. Priming with TGFβ3 upregulated TGFBI transcription in murine MSCs and human MSCs and increased TGFBI secretion in human MSCs. Moreover, incubation of osteoarthritis-like mouse chondrocytes with TGFβ3-primed murine MSC-conditioned media significantly upregulated the expression of chondrocyte anabolic markers but did not change the expression of catabolic and inflammatory factors [131]. BMPs are a subfamily of the TGF-β superfamily that participate in the induction of bone and cartilage formation. BMP6 enhanced chondrogenesis of MSCs [132]. As key transcription factors for pluripotency and self-renewal, the overexpression of Nanog and Oct4 also enhanced chondrogenic reported that cell therapy by using MSCs after neurogenic differentiation and maintained MSCs properties in various culture conditions [133]. Stromal cell-derived factor-1α has been detected to promote stem cell migration and homing [134].

### Non-coding RNAs

Non-coding RNAs are functionally complex and are implicated in many crucial biochemical and cellular processes such as cell communication, inflammation, exosome biogenesis, tissue repair, regeneration, and metabolism. This wide distribution of biological activities confers on MSC-Exos the potential to elicit diverse cellular responses and interact with many cell types. MiR-410 is a key regulator of MSC chondrogenic differentiation and directly targets Wnt3a triggering the Wnt signaling pathway [135]. Moreover, miR-127-3p from BM-MSCs inhibited cadherin-11 in chondrocytes, thereby blocking the Wnt/β-catenin pathway [96]. The miR-155-5p in synovial fluid-derived MSC-Exos promoted proliferation and migration, suppressed apoptosis, and enhanced ECM secretion of osteoarthritic chondrocytes. Further, overexpression of Runt-related transcription factor 2 partially reversed the effect of the synovium-derived MSC-155-5p-Exos on osteoarthritic chondrocytes [136]. MiR-135b promoted M2 polarization of synovial macrophages through targeting MAPK6 [117]. Additionally, miR-361-5p was verified to inhibit the NF-κB signaling pathway [137]. Synovium-derived MSC-extracellular vesicle-encapsulated miR-31 ameliorates KOA via the lysine-specific demethylase 2A/E2F transcription factor 1/pituitary tumor transforming gene 1 axis [91]. The upregulation of miR-143 and miR-124 in cellular and mouse OA models treated with Exos remarkably restored the normal expression of NF-κB and Rho Kinase 1 pathways [138]. Human AT-MSCs-Exos inhibited cell apoptosis, enhanced matrix synthesis, and reduced the expression of catabolic factors via the mTOR signaling pathway. MiR-100-5p decreased the luciferase activity of the mTOR 3′-untranslated region [97]. Hu et al. revealed that miR-365 expression was activated by chondrogenic induction in both MSCs from the osteoarthritis model and BM-MSCs [139]. Additionally, some micro-RNAs protect the cartilage, such as miR-26a-5p targeting phosphatase and tensin homolog, miR-26a-5p targeting prostaglandin-endoperoxide synthase 2 [111, 112], miR-136-5p targeting E74-like factor 3 [101], and miR-520d-5p targeting histone deacetylase 4 [140]. Most reported non-coding RNAs were detected in the Exos, and detailed mechanisms are presented in Table 2.

**Table 2:** Mechanisms of non-coding RNA in mesenchymal stromal cell-based therapy for knee osteoarthritis

Source	Target	Amount	Axis/signaling pathway	Function	References
Human AT-MSCs	In vitro	400 µg/mL	miR-145 and miR-221	Downregulated the expression of pro-inflammatory markers IL-6, NF-κB, and TNF-α, while upregulated the expression of the anti‐inflammatory cytokine IL‐10	[109]
Human OA cartilage-derived MSCs and BM-MSCs	Mice	NR	miR-365	Activation of aggrecan and collagen type 2a1 gene expression. MiR-365 expression was activated by chondrogenic induction in both OA-MSCs and BM-MSCs	[139]
Human BM-MSCs-Exos	In vitro	NR	miR-520d-5p/HDAC1	MiR-520d-5p promoted MSCs chondrogenesis and regulates chondrocyte metabolism through targeting HDAC1	[140]
Human BM-MSCs	In vitro	NR	miR-410/Wnt3a	MiR-410 was elevated during TGF-β3-induced chondrogenic differentiation of MSCs, and regulated the Wnt signaling pathway	[135]
Rat BM-MSCs-Exos	Rat	NR	miR-9-5p/syndecan 1	Anti-inflammatory and chondroprotective effects of BM-MSC-derived exosomal miR-9-5p on KOA via regulation of syndecan 1	[110]
Human BM-MSCs-Exos	Rat	250 ng/5 µL	miR-26a-5p/Cox2	Human BM-MSC-Exos overexpressing miR-26a-5p serve as a repressor for damage of synovial fibroblasts via Cox2 in KOA	[112]
Human SMSCs-Exos	Rat	30 µL, 10^11^ particles/mL	miR-26a-5p/PTEN/IL-1β	SMSC-exos enhanced IL-1β-induced cell proliferation, whereas inhibited apoptosis and inflammation. MiR-26a-5p targeted PTEN, for which overexpression spoiled the protection of exosomes against IL-1β-induced cell damage	[111]
SMSCs-Exos	Mice	5 µL	miR-31/KDM2A/E2F1/PTTG1	SMSC-Exos and Exos from miR-31-overexpressed SMSCs alleviated cartilage damage and inflammation in KOA in vivo	[91]
Human AT-MSCs-Exos	Mice	10 µL, 10^10^ particles/mL	miR-100-5p/mTOR	The level of miR-100-5p decreased the luciferase activity of mTOR 3′UTR, while inhibition of miR-100-5p could reverse the MSC-Exos-decreased mTOR signaling pathway	[97]
Rat BM-MSCs-Exos	Nude mice	20 µg	miR-127-3p/ CDH11/Wnt/β-catenin	MiR-127-3p targeted CDH11 and over-expressed CDH11 in chondrocytes weakened the therapeutic effect of exosomes. IL-1β treatment resulted in the activation of the Wnt/β-catenin pathway in chondrocytes	[96]
Rat BM-MSCs-Exos	Rat	100 µL, 10^11^ particles/mL	miR-135b/MAPK6	MiR-135b promoted M2 polarization of synovial macrophages through targeting MAPK6	[117]
Rat MSCs-Exos	Rat	100 µL, 10^11^ particles/mL	miR-135b/Sp1/TGF-β1	TGF-β1 stimulation enhanced miR-135b expression in MSC-exosomes, and MSC-exosomes-derived miR-135b increased the cell viability of C5.18 cells via downregulated Sp1 expression	[92]
Human BM-MSCs-Exos	Mice	100 µL, 10^11^ particles/mL	miRNA-136-5p/ELF3	An increased ELF3 expression and reduced miR-136-5p expression were detected in the clinical samples of traumatic OA cartilage tissues. BM-MSC-derived exosomal miR-136-5p could promote chondrocyte migration in vitro and inhibit cartilage degeneration in vivo	[101]
Human AT-MSCs-Exos	Mice	NR	miR-124/NF-κB and miR-143/ ROCK1/TLR9	MiR-143 and miR-124 inhibited the expression of NF-κB and ROCK1 in OA cells. In addition, the 3’ UTRs of NF-κB and ROCK1 were proven to contain the binding sites for miR-143 and miR-124, respectively	[138]
Rat BM-MSCs-Exos	Rat	200 µg	miR-216a-5p/JAK2/STAT3	Hypoxic-Exos promoted the proliferation and migration of chondrocytes and inhibited their apoptosis by targeting functional miR-216a-5p to chondrocytes and then downregulating JAK2. In addition, HIF-1α induces hypoxic BM-MSCs to release Exos	[93]
Human BM-MSCs-Exos	Rat	2 µg	miR-361-5p/DDX20/NF-κB	MiR-361-5p was verified to directly target DDX20. Additionally, human BM-MSC-Exos-transferred miR-361-5p alleviates chondrocyte damage and inhibits the NF-κB signaling pathway	[137]
Human SMSCs-Exos	BALB/C mouse	30 µL, 10^11^ particles/mL	miR-155-5p/Runx2	The SMSC-155-5p-Exos prevented KOA. Overexpression of Runx2 partially reversed the effect of the SMSC-155-5p-Exos	[136]
MSCs-Exos	In vitro, co-culture with mouse chondrocytes	NR	circRNA_HIPK3/miR-124-3p/MYH9	MSCs-Exos overexpressing circHIPK3 improved IL-1β-induced chondrocyte injury. Mechanistically, circHIPK3 could directly bind to miR-124-3p and subsequently elevate the expression of the target gene MYH9	[143]
Human MSCs	In vitro	NR	lncRNA HOTAIRM1-1/miR-125b/ BMPR2; JNK/MAPK/ERK pathway	HOTAIRM1-1 was downregulated in KOA cartilages and may inhibit MSCs viability, induce apoptosis, and suppress differentiation via regulating miR-125b/BMPR2 axis JNK/MAPK/ERK pathway may be a possible downstream mechanism to mediate the role of HOTAIRM1-1 in OA development	[146]
Human BM-MSCs-Exos	In vitro	NR	lncRNA HOTTIP/miR-455-3p/CCL3	HOTTIP negatively regulated miR-455-3p and increased CCL3 levels in human chondrocytes	[147]
Human AT-MSCs	In vitro	NR	circRNA_ATRNL1/miR‐145‐5p/SOX9	Circ_ATRNL1 regulated the promotion of SOX9 expression to promote chondrogenic differentiation of human AT-MSCs mediated by miR‐145‐5p	[141]
Human BM-MSCs-Exos	Mice	10 µL, 500 µg/mL	circRNA_0001236/miR-3677-3p/Sox9	Exosomal circRNA_0001236 enhanced the expression of Col2α1 and SOX9, but inhibited MMP13 in chondrogenesis via targeting miR-3677-3p and Sox9	[142]
Human BM-MSCs	In vitro	NR	lncRNA GRASLND	Silencing of lncRNA GRASLND resulted in lower accumulation of cartilage-like extracellular matrix, while GRASLND overexpression significantly enhanced cartilage matrix production	[144]
Human SMSCs	In vitro	NR	lncRNA MEG3/EZH2-mediated H3K27me3/TRIB2	LncRNA MEG3 regulated chondrogenic differentiation by inhibiting TRIB2 expression through EZH2-mediated H3K27me3	[145]
Human and mouse MSCs	In vitro	NR	lncRNA EPB41L4A‐AS1 and lncRNA SNHG7/miR‐146a	MiR‐146a significantly inhibited BM-MSCs proliferation partly interacting with lncRNA EPB41L4A‐AS1 and lncRNA SNHG7	[148]
Human BM-MSCs-Exos	In vitro	NR	lncRNA LYRM4-AS1/GRPR/miR-6515-5p	IL-1β significantly decreased cell viability, promoted apoptosis, and upregulated the expression of MMP3, AKT, and GRPR, while Exos reversed the changes	[149]

*OA* Osteoarthritis, *KOA* knee osteoarthritis, *MSCs* mesenchymal stromal cells, *BM* bone marrow, *AT* adipose tissue, *Exos* exosomes, *NR* not reported, *IL* interleukin, *NF-κB* nuclear factor-kappaB, *TNF-α* tumor necrosis factor-α, *TGF-β* transforming growth factor-β, *UTR* untranslated regions of mRNA, *CDH11* cadherin-11, *SMSCs* synovial-derived mesenchymal stromal cells, *Runx2* Runt-related transcription factor 2, *MAPK* mitogen-activated protein kinases, *DDX20* Asp-Glu-Ala-Asp (DEAD)-box polypeptide 20, *ROCK1* Rho-associated kinase 1, *TLR9* Toll-like receptor 9, *mTOR* mechanistic target of rapamycin, *JAK2* Janus kinase 2, *STAT3* signal transducer and activator of transcription 3, *PTEN* phosphatase and tensin homolog, *HDAC1* histone deacetylase 1, *Cox2* cyclooxygenase-2, *ELF3* E74-like factor 3, *SOX9* sex-determining region of Y chromosome-box transcription factor 9, *Col2α1* α-1 chain of procollagen type 2, *MMP* matrix metalloproteinase, *MYH9* myosin heavy chain 9, *TRIB2* tribbles homolog 2, *BMPR2* bone morphogenetic protein receptor 2, *JNK* p38 and c-jun N-terminal kinase, *ERK* extracellular signal-regulated kinase, *CCL3* macrophage inflammatory protein 1-α, *AKT* protein kinase B, *GRPR* gastrin-releasing peptide receptor

Moreover, circle-RNAs and long non-coding RNAs play vital roles in micro-RNAs interaction and show abnormal expression in osteoarthritis, which may be an important target for regulating osteoarthritis and for drug treatment. These RNAs regulate the progress of KOA by completing with micro-RNAs or other non-coding RNAs, that is called the ceRNA regulatory network, such as circRNA_ATRNL1 targeting miR‐145‐5p [141], circRNA_0001236 targeting miR-3677-3p [142], circRNA_HIPK3 targeting miR-124-3p [143], lncRNA GRASLND [144], lncRNA MEG3 targeting EZH2-mediated H3K27me3 [145], lncRNA HOTAIRM1-1 targeting miR-125b [146], lncRNA HOTTIP targeting miR-455-3p [147], lncRNA EPB41L4A‐AS1 and lncRNA SNHG7 targeting miR‐146a [148], and lncRNA LYRM4-AS1 targeting miR-6515-5p [149].

## The physical situation on MSC-based cells

The external physical situation could affect the cartilage and MSCs phenotype, such as conditions with hypoxia, hydrostatic pressure, compression, or magnetic fields. Nonetheless, the standard culture systems of the external physical situation have not been well-established yet and mechanisms are unclear.

Several studies have determined the application of low oxygen tension or hypoxia in MSC chondrogenesis and culture. In the presence of IL-1β, a significant in glycosaminoglycan, as a measure of proteoglycan levels, collagen, and water content, was observed under hypoxic condition (2% O_2_, 5% CO_2_, 93% N_2_) [150]. Moreover, the mRNA expression of Col-II and aggrecan was upregulated in chondrocytes co-cultured with BM-MSCs under hypoxia (5% CO_2_ and 95% N_2_), and DNA methylation of the SOX9 promoter was significantly decreased under hypoxia [90]. Low oxygen tension (5%) was observed to promote ECM production by chondrocytes and enhanced the chondrogenesis of AT-MSCs compared to that cultured in normal condition [151]. Grayson and colleagues showed that under a 2% O_2_ hypoxia, the expression of stromal cell genes Oct-4 and Rex-1 was upregulated [152].

Magnetic fields have been reported to enhance the chondrogenic differentiation of MSCs. Pulsed electromagnetic fields drastically promoted chondrogenesis by a specific hydrogel with high expressions of Col-II, aggrecan, and SOX9 genes [153]. Besides pulsed electromagnetic fields, a static magnetic field with 0.4 T was demonstrated to produce a strong chondrogenic differentiation response after 14 days of culturing through the TGF-β pathway [154]. Further, the presence of electromagnetic fields could partly replace the addition of TGF-β3, while the efficacy of chondrogenesis was statistically increased in the culture system. In TGF-β3-treated pellets, a further significant increase of 72.7% in aggrecan gene expression was induced by electromagnetic fields at 5 weeks [155].

Ultrasound-targeted microbubble destruction has been confirmed to increase the homing of transplanted MSCs to targeted organs. Stromal cell-derived factor-1α, as an important role in BM-MSCs migration, was loaded in microbubble. The number of migrated cells was higher when loaded microbubble under the guidance of ultrasound that the ultrasonic irradiation conditions of duty ratio 10%, intensity 1 W/cm^2^, time 30 s [134].

## Clinical application

The initial injection of MSC-based therapy was in 2008 [156], and a total of 23 non-case report studies are reported to apply MSC-based therapy for KOA since then. Thirteen of them were designed as randomized controlled studies, although with heterogeneity in sources cell, preparation methods, and dosage of MSCs. No study using MSC-Exos in KOA has been revealed yet. BM was the most frequently used source of MSCs (13/23 studies; 57%) [157–169], AT was used in 7 trials (30%) [18, 19, 170–174], and umbilical cord was used in two in two trial (9%) [15, 175], and one study used MSCs from placenta (4%) [176]. In 12 trials in phase I, the different dosages were compared. The control interventions in the rest trials were hyaluronic acid injection in five trials [15, 157, 160, 163, 175], PRP injection in four trials [161, 167, 168, 172], saline injection in four trials [19, 163, 164, 176], total knee arthroplasty in one study [162], and conservational treatment [18]. Combined therapy included total knee arthroplasty [162], PRP [161], PRP with arthroscopic debridement [172], and hyaluronic acid [160]. Most included individuals had grade II–III of Kellgren-Lawrence. Table 3 summarizes the study characteristics.

**Table 3:** Characteristics of clinical trials about mesenchymal stromal cell-based therapy for cartilage regeneration in knee osteoarthritis

Design	Sample size	Source	Dosage (cells)	Control intervention	Phase of trial	K-L grade	Outcomes	Follow-up	References
RCT	30	BM-MSCs	4 × 10^7^	HA	II	II-IV	MRI, WOMAC, VAS, Lequesne index	12 months	[157]
RCT and observational study	18	AT-MSCs	1, 2, 5 × 10^7^	None	I/IIa	> II	AE, WOMAC, NRS, SF- 36, MRI	24 months	[170]
Observational study	15	BM-MSCs	4 × 10^7^	None	I/II	II-III	AE, WOMAC, VAS, Lequesne index SF- 36, MRI	12 months	[158]
Observational study	18	AT-MSCs	2, 10, 50 × 10^6^	None	I	III-IV	AE, WOMAC, VAS, KOOS	6 months	[171]
Observational study	12	BM-MSCs	4 × 10^7^	None	II	II-IV	MRI, WOMAC, VAS, SF-36	12 months	[159]
RCT	26	UC-MSCs	2 × 10^7^	HA	I/II	I-III	MRI, WOMAC	12 months	[15]
RCT	24	AT-MSCs	1 × 10^8^	Saline	IIb	II-IV	MRI, WOMAC, KOOS	6 months	[19]
RCT	30	BM-MSCs	1, 10 × 10^7^	HA	I/II	> II	MRI, WOMAC, VAS	12 months	[160]
RCT	60	BM-MSCs	1 × 10^8^	PRP	II	> II	MRI, WOMAC, VAS	12 months	[161]
Observational study	25	AT-MSCs	1.89 × 10^6^	PRP, arthroscopic debridement	II	I-III	Lysholm, Tegner activity scale, VAS	16 months	[172]
RCT	20	Placenta-derived MSCs	5–6 × 10^7^	Saline	II	II-IV	VAS, KOOS, ROM, MRI	6 months	[176]
Observational study	18	AT-MSCs	1, 5, 10 × 10^7^	None	I/II	> II	WOMAC, MRI, arthroscopy	6 months	[173]
RCT	140	BM-MSCs	1.56 × 10^4^	TKA	III	II-IV	Radiographs, MRI	15 years	[162]
RCT	60	BM-MSCs	25, 50, 75, 150 × 10^6^	HA	I/II	II-III	WOMAC, VAS	12 months	[163]
RCT	30	AT-MSCs	1 × 10^8^	Conservative management	II	II-III	AE, MRI, KOOS, WOMAC, NRS	12 months	[18]
RCT	43	BM-MSCs	4 × 10^7^	Saline	I/II	II-IV	VAS, WOMAC	6 months	[164]
Observational study	29	UC-MSCs	1 × 10^7^	HA	II	I-II	WOMAC	6 months	[175]
Observational study	4	BM-MSCs	8–9 × 10^6^	None	I	NR	VAS, X-ray, activities	12 months	[165]
Observational study	12	AT-MSCs	5 × 10^7^	None	I	NR	AE, MRI	12 months	[174]
Observational study	12	BM-MSCs	1, 10, 50 × 10^6^	None	I/IIa	III-IV	ROM, KOOS, WOMAC, MRI	12 months	[166]
RCT	18	BM-MSCs	NR	PRP	II	II-IV	KOOS, ROM	12 months	[167]
RCT	57	BM-MSCs	NR	PRP	II	II-IV	KOOS, ROM	12 months	[168]
Observational study	12	BM-MSCs	6 × 10^7^	None	I	II-III	AE, KOOS, MRI	24 months	[169]

*RCT* Randomized controlled trial, *MSCs* mesenchymal stromal cells, *BM* bone marrow, *AT* adipose tissue, *NR* not reported, *AE* adverse event, *NRS* numerical pain rating scale, *VAS* visual analog scale, *WOMAC* Western Ontario and McMaster Universities Osteoarthritis Index, *SF-36* short-form 36 health survey questionnaire, *KOOS* Knee Injury and Osteoarthritis Outcome Score, *MRI* magnetic resonance imaging, *TKA* total knee arthroplasty, *HA* hyaluronic acid, *PRP* platelet-rich plasma, *K-L* Kellgren-Lawrence

### Dosage of MSCs

The single injection dosage ranged from 1.56 × 10^4^ to 1 × 10^8^ cells, and the most wildly proved dosage was 5 × 10^7^ cells. Repeated injections or high dosage showed superiority than single injection or low dosage. Matas et al. clarified that repeated umbilical cord-derived MSCs (2 × 10^7^ cells, every half year) treatment showed better improvements in pain and function than receiving injection only once at 1-year follow-up for individuals with KOA [15]. Moreover, Lamo-Espinosa et al. reported a high dosage with 1 × 10^8^ BM-MSCs together with hyaluronic acid resulted in a larger clinical and functional improvement [160]. In addition, Chahal et al. found lower cartilage catabolic biomarkers and MRI synovitis in participants with higher doses [166] and the effects were maintained until 2-year follow-up [177].

### Safety of MSC-based therapy

In general, MSC-based therapy is safe with mild adverse events. The most common adverse events were transient arthralgia, swelling of joints after local injection [171], and low back pain [158], which were mild to moderate and were usually spontaneously relieved within 7 days without special treatment or controlled with ibuprofen [159]. Song et al. reported one patient experienced mild edema and cramps of bilateral lower extremities that were relieved in 21 days without treatment [170]. Pers et al. demonstrated one severe adverse event that one patient with hypertension and hyperlipidemia experienced unstable angina pectoris without creased cardiac markers 3 months after AT-MSCs injection [171]. Adverse events were predominant in the high doses (> 5 × 10^7^ cells) [163].

### Effects on structure, pain, activities, and quality of living

Intra-articular injections of MSCs improved structure, pain, the function of the knee joint, rendering them a promising novel treatment for KOA. Besides these, this therapy had potential in activities, such as climbing the stairs and walking, and inflammatory factors. These benefits may last for several years, even be more apparent after months. Orozco et al. demonstrated an average 27% decrease of poor cartilage areas in severe KOA [159]. For MSCs-treated patients, Vega et al. [157] and Dilogo et al. [175] reported cartilage quality improvements that were quantified by T_2_ relaxation measurements. Although Lee et al. thought there was no improvement of cartilage at 6 months in the MSCs group, whereas the defect in the control group was increased [19]. Radiological, arthroscopic, and histological measures consistently demonstrated decreased deterioration by regeneration of hyaline-like articular cartilage [173]. For pain and function, Soler et al. [158] revealed a relevant pain relief since day 8 and maintained after one year. Khalifeh et al. clarified improvement in range of motion of the knee joint after MSCs injection was significant between the 2-week and 24-week follow-up [176]. The walking time without pain improved [165]. Although knees deteriorate gradually, they were still better than at baseline at 5 years [178]. Pro-inflammatory monocytes/macrophages [166] and IL-10/12 levels [168] decreased in the synovial fluid after MSCs injection.

Compared with ongoing conventional conservative management, AT-MSCs showed clinically significant pain and functional improvement after one year [18]. Moreover, greater improvements in Western Ontario and McMaster Universities Osteoarthritis Index (WOMAC) total score, WOMAC pain, and physical function were recorded than saline [164]. There were greater improvements in the pain, function, daily living activities, and sports and recreational activities subscales in people receiving MSCs therapy than PRP, although less than the combination of two therapies [167].

## Future directions

Based on the current application states, different cell sources have been clarified in the clinic by phase I/II studies, while no MSC-Exos are used. Therefore, a standard for MSCs therapy in KOA is required, which includes cell selection, authentication (phenotypic analysis and multipotent differentiation potential, especially distinguish with progenitor cells), culture or expansion methods, dosages, and rehabilitation program after injection. Second, Exos are good cargo and have potential in clinic application. The contained specific non-coding RNAs are important and may have an essential influence on the therapeutic effects. LncRNAs and circRNAs in Exos and the safety and doses of Exos need large research, which will be the aim of future clinical trials. Third, previous researchers have focused on the influence of physical situations on MSCs, while the mechanisms are still unclear. In addition, no study reported the changes of MSCs-Exos, RNA, or DNA sequence after physical exposure, which might be an unexpected trend. Although we merely discuss biological materials in MSC-based therapy, scaffold-assisted grafts or complex 3D hybrid tissues of MSCs or Exos with or without electromagnetic fields are interesting trends for severe KOA. At last, besides cartilage regeneration, functional subchondral bone regeneration also has a significant impact on KOA treatment.

## Conclusions

MSCs and MSC-exosomes, as new therapeutic methods for KOA, showed unique advantages. The selection of different origins of MSCs may be inconsistent based on the research goal and the phenotype exhibits various characteristics. MSCs and the derived exosomes carried out various functions in the treatment of KOA which include of increase of chondrogenesis, proliferation of chondrocyte, reduction of apoptosis, maintenance of autophagy, regulation synthesis and catabolism of the ECM, regulation of immune response, inhibition of inflammation, monitoring the mitochondrial dysfunction, and the paracrine effect. These functions were partly demonstrated through several biological pathways or axis, such as NF-κB, MAPK, ROS, and mTOR pathways. Exosomes are primary mediators of intercellular communications especially by transferring non-coding RNAs to adjacent cells or remote cells. Different physical conditions (hypoxia, magnetic fields, and ultrasound) have been studied to enhance the functions in MSC-based therapy experiments. This review has presented the evidence for MSC-based therapy as a new approach to the cell-free treatment of KOA. However, a standard for MSC-based therapy in KOA is required.

## Data Availability

Not applicable.

## Abbreviations

KOA: Knee osteoarthritis
ECM: Extracellular matrix
MSCs: Mesenchymal stromal cells
Exos: Exosomes
BM: Bone marrow
AT: Adipose tissue
CD: Cluster of differentiation
TGF-β: Transforming growth factor-beta
SOX9: Sex-determining region of Y chromosome-box transcription factor 9
TLR: Toll‐like receptor
TNF‐α: Tumor necrosis factor‐α
MMPs: Matrix metalloproteinases
TIMPs: Tissue inhibitors of MMPs
Col: Collagen
IL: Interleukin
NF-κB: Nuclear factor-kappaB
MAPK: Mitogen-activated protein kinase
ERK: Extracellular regulated protein kinases
AKT: Protein kinase B pathways
ROS: Reactive oxygen species
GSK-3β: Glycogen synthase kinase-3β
CDK: Cyclin-dependent kinase
TGFBI/BIGH3: TGF-β-induced gene product-h3
BMPs: Bone morphogenetic proteins
WOMAC: Western Ontario and McMaster Universities Osteoarthritis Index
PRP: Platelet-rich plasma
